# Abdominal Operations

**Published:** 1907-08-31

**Authors:** J. H. Dauber

**Affiliations:** Gynæcologist to the Hospital for Women, Soho


### Hospital Clinics.

### ABDOMINAL OPERATIONS.

### A Consideration of Some Factors which Contribute to Their Success.

By J. H. DAUBER, M.A., M.B., B.Ch.(Oxon.), Gynæcologist to the Hospital for Women, Soho.

It is obvious that a surgeon who will only operate upon selected cases will, other things being equal, obtain better results than the man who operates upon each case as it comes. On this account it is difficult to compare operators one with another. One will accept risks which another will decline, and so mere statistics are of little value as a criterion of surgical merit. It is only by direct observation that a man's work can be judged.

Old people, in my experience, bear abdominal operations well, provided the duration of their performance is not too long. Cases of malignant disease stand in a class by themselves, and are always attended with greater risks. The mortality amongst them is proportionally much higher than in non-malignant cases. Operation upon them often has to be very free, involving all organs, glands, and tissues that have been actually or probably invaded by the cancer cells. The subjects of malignant disease are often aged and debilitated by pain and septic absorption. They therefore need especial care.

Most patients previous to operations are in a state of nervous tension and very impressionable. Therefore every influence about them should be optimistic. Nurses should refrain from discussing similar or any operations with their patients. All nurses in my experience forget that though speech is silvern, silence is golden. As little as possible of the preparations for operation should be subjected to the view of the patient.

The question of the best anæsthetic to be employed is too large to be entered into. If there is any suspicion of bronchial trouble I ask that chloroform may be used. That anæsthetic is generally to be preferred to which the anæsthetist is most accustomed, and it is well to give him a free hand. The surgeon has his own hands sufficiently full without concerning himself with the administration of the anæsthetic. It is important, however, to him that complete relaxation of the abdominal muscles should be maintained throughout.

Oral asepsis must, previous to operation, receive careful attention. Carious teeth and purulent gums are often productive of toxæmic symptoms, and parotitis and parotid abscess are not the least of the troubles that may be caused thereby. It may be necessary to requisition the services of a dentist, though patients often resent this with unusual vigour. At all events the nurses cannot be too assiduous in their endeavours to keep the buccal cavity in a clean and healthy condition.

Before operation the skin area prepared by compresses is, according to my view, not always large enough. The course of operations does not always run smooth. Emergencies arise, more handling of the patient becomes necessary than was anticipated—therefore let a large area be prepared. The same applies to the preparation of the skin when the patient is actually on the operating table. I generally cleanse with (1) ether soap; (2) methylated spirit; and (3) 1 in 2,000 biniodide in spirit, using all three freely; groins, loins, lower ribs, and pubes being included.

Patients should not be kept too long in hospital before being operated upon. The tension of a week in the wards is often too much for them, and they leave the hospital unhappy and unoperated upon. On the other hand, no patient with an inflammatory condition in the pelvis should be operated upon—unless it is an urgency case—for a few days after admission.

I have noticed that patients are often subjected to too cold an atmosphere before entering the operating room. Continuous warmth is essential from the moment the patients leave their beds until their return. The anæsthetising room should be as warm as the theatre, and the operating table should contain hot water apparatus. It is a mistake, in my opinion, for patients to be returned into a general ward while intoxicated with the anæsthetic. To have a shouting, vomiting woman on one or both sides for some hours frightens and often completely upsets a nervous woman, quite unused to hospital life, and herself expecting to be operated upon in a day or two. I have seen this and have been vexed and pained by it. There should be a separate room for people coming round after operations; they should not be let loose upon a peaceful ward until quiet. But this room, or any room for the reception of patients after operation, must be warm. If warmth is essential before operation it is doubly so afterwards. Patients are often sent through draughty corridors, placed in cold lifts, or put in wards with open windows when suffering from the shock of operations. It is not always ether that is the cause of subsequent bronchitis. Remember, too, that the anæsthetising apparatus should be disinfected between each case. Death from septic bronchitis has sometimes resulted from neglect of this precaution. To maintain an equable warmth and obviate shock the practice of wrapping the patient in cotton wool, with the exception of the operation area, is excellent. I need not remind you that special warm wool surgical stockings are sold, and that every nurse knows how to make a pneumonia jacket. Cotton wool for thighs and arms may be useful in addition in severe cases. These warm things must not be parted with too hastily subsequently, but gradually discontinued. Patients like them moreover, and find them comforting. Before operation patients should never be allowed to get too "low." No one should be more than three and a half hours on an empty stomach while waiting for operation. Too often in hospital all the patients are prepared as if to be operated upon simultaneously, whereas there may be a space of two, three, or even more hours between the first and last cases.

Asepsis.

With regard to the operation itself, I consider asepsis in every minute detail of superlative and paramount importance. Everyone who comes in contact with the patient should wear sterilised overalls. Boiled gloves are indispensable for nurses and assistants alike, as well as for the operator. In short, everything that can come near the patient should be sterilised, by heat if possible. From every point of view thermal agencies are preferable to chemical in effecting asepsis. For this reason I always use silk, or thread, or something that I can see boiled myself, in preference to catgut or other material that I may have to take on trust. Marine sponges are not to be thought of for this reason. Armlets of india-rubber should be worn by both operator and assistant. I have often seen the surgically unclean arm of either or both come into contact with instruments or ligatures, in spite of the wearing of gloves. The armlets should reach to the sterilised overalls.

In respect of asepsis one must for ever be on one's guard, and I have known men who have written volubly on asepsis commit unwittingly glaring mistakes. The older school seem quite incapable of assimilating modern doctrines into their practice. Nurses, too, are very weak in this direction. It is not an uncommon experience to go to a surgical home taking gloves with one for the chief nurses, and to find them subsequently, while still wearing their gloves, prepared to touch all and sundry articles, sterilised or unsterilised, such as taps, jugs, bedding, and the like; some of them think nothing of opening or shutting a door or window in them. All should remember that there is no more perishable article, in an aseptic sense, than one that has been sterilised. Almost "a breath can mar it." It will be said, "Oh yes, but all this is not necessary; excellent results can be and are obtained without all this fuss every day." To this I would reply, Many a cranky ship has crossed the Atlantic in safety times and again, but who of us would not prefer to make the voyage in a ship of the highest class, notwithstanding this fact? Our patients entrust their lives to us, and it is nothing less than treachery on our part if we betray that trust by carelessly neglecting to eliminate every possible risk.

Drainage.

Details in operating I have not now the space to discuss. Personally, I very rarely employ drainage unless cutting down directly upon an abscess merely for that purpose. I have often left large areas of pyogenic membrane, that I could not remove, in the abdominal cavity without the slightest ill result—sometimes I have merely swabbed it carefully, at others copiously irrigated. "Clean well and close well" is my motto. In exploratory operations a fruitless incision in one place, and then another somewhere else, possibly with like result, is inartistic to say the least of it, if not dangerous, and says little for our diagnosis. Large incisions are now the fashion, and rightly so. Sewing up the abdominal wall in its various layers has robbed ventral hernia of its terrors. We rarely see it except in some cases where drainage has been employed. The length of an incision is unimportant; it is as well to see as to feel what one is doing, and easier to work through a large hole than a small one. An incision that only admits two fingers generally has to be enlarged before what is necessary can be accomplished. Free incisions and the Trendelenburg position when necessary commend themselves to me.

Lightness of touch and gentleness are all important. Some excellent surgeons whose technique is perfect yet fail in obtaining the best results. Such often have strong, heavy hands, and use them with a strength that is detrimental to delicate tissues. It is the oft-handled bruised tissues that succumb to toxæmic attack, not the gently touched, briefly exposed, and unchilled.

Long and Short Operations.

If there is one thing I believe in it is rapidity in operating. A rapid operator can almost afford a little laxity in aseptic technique, though I say this under my breath. It is customary to say, "the rapid operating of Fergusson would be out of place to-day—with anæsthesia we can operate at our leisure." Not at all—every minute is of value now as it was then. Not a second should be lost from first to last. Be ready to make your incision the moment the anæsthetist gives the word. A harassing complication, such as one involving a resection of gut, may occur at any moment, when the man with time in hand will score heavily. Time is on the side of death, not on that of the surgeon. Every operation should be completed within an hour, if possible. At the end of that time I should like a bell to be tolled continuously, or other means taken to remind the operator that he was within the danger zone. I say this deliberately—most patients can stand an hour well, but after that every additional five minutes is fraught with more and more risk. If there is much hæmorrhage, then there is the more need for haste. A long operation with considerable hæmorrhage is a deadly risk to any patient, and to the old especially. After long operations every precaution must be taken to keep up warmth and the circulation. Hypodermic, rectal and saline injections all have their place. In my opinion direct intra-venous saline injections are unrivalled by other methods, but it needs a little skill and practice quickly to dissect out the vein and transfuse into it, whereas anyone can administer a rectal or sub-cutaneous injection.

Many assistants at an operation are so many additional sources of risk. At some general hospitals I have seen three or four dressers assisting the operator at an abdominal operation, in addition to the house surgeon, each one holding a retractor or forceps or other little tool, and between whiles not knowing where to put his hands—and we know from Dr. Watts who it is that "finds some mischief still for idle hands to do." This is not quite so bad as the dear old matron, the despair of a certain hospital staff, who, even when put into surgical gloves, would shake hands in them if any old student of her acquaintance entered the theatre; after which she would go on with her office of handing swabs and bowls.

After Treatment.

Now a word as to after treatment. Rectal injections of normal saline are invaluable for relieving thirst, and may be repeated *ad libitum* at all times. If vomiting is troublesome, or shock pronounced, nutrient enemata are excellent to supplement or assist oral feeding. Patients suffer much from thirst, and to withhold water is cruel and needless.

Everything has become much simplified of late years in the after treatment of these cases. Patients may be moved gently to a certain extent from side to side, and so spared that painful backache which otherwise is so distressing to them. A sixth of a grain of morphia hypodermically never does any harm, but more often it is not needed. Flatulent distension of the bowels is still the great trouble after cœliotomy. The rectal tube should be freely used in these cases, and purgatives and rectal injections must be resorted to unremittingly until the bowels act; after that patients may eat whatever they can.

Points About the Diet.

I am quite convinced that not nearly enough attention is given to the culinary arrangements, either in hospitals or nursing homes. I have often been disgusted with the way in which the food is served to patients in hospitals. In the matter of surgical dressings and appliances, nothing is too good for them; but when it comes to the way in which their food is served to them, then it is another affair altogether. Some years ago I was staying a few weeks in one of the chief towns in the south of Spain, and visited the military hospital there. The commandant, a most courteous old colonel, showed me everything with the utmost kindness, but he explained to me that if I noticed many deficiencies it was because the Government would not supply them, and, moreover, the new methods had come in since his time, and he did not mind them much; but what he did think was really important was that the patients should be *well fed*, and so I was taken to the kitchens and was made to taste all the dishes—the soup and the wine, etc.—and was asked my opinion of them. I pronounced them excellent, as undoubtedly they were. There are other things that make for success in surgery besides the latest type of steriliser. After operations, when convalescing, the appetites of patients want coaxing and tempting, no matter how low the social class to which they belong. How often is the food sent away because it is served untemptingly, the meat cut too thick, or cold, underdone or overdone, or generally unappetising? It is the same in a large percentage of the surgical homes. The commissariat is weak to the last degree, and the culinary art often almost unknown. Both the quality and quantity of the food supplied are inadequate, and complaints are heard on all sides. If, as Napoleon said, " a soldier marches on his belly" certainly a patient convalesces on nothing else.

A certain most excellent teacher of surgery was always reminding us as students, when speaking of fractures, that we must restore the function of a broken limb, and not be content with merely getting the ends to unite in good position. And so with abdominal work. Do not forget that to some an operation and the subsequent convalescence is a great ordeal, and taxes their powers exceedingly. It may be three to six months before they are able to resume their usual occupations. Our work is not merely an exalted form of human carpentry. Surgery does not consist alone of manipulative dexterity and operative skill. Judgment, based on knowledge and experience, is still the master quality in our equipment, and many side issues have to be considered and disposed of by us before our patients are completely restored to health.

All I have said has, I am aware, been said, and said better, before. I have doubtless omitted many essential facts, but I have endeavoured to emphasise some of the points which in my judgment are important in abdominal and pelvic operations.

